# Ordered Mesoporous Ceria and Cerium Gadolinium Oxide Prepared by Vacuum-Assisted Nanocasting

**DOI:** 10.3390/nano14080651

**Published:** 2024-04-09

**Authors:** Troy A. Dougherty, Richard T. Baker

**Affiliations:** EaStChem, School of Chemistry, University of St Andrews, St Andrews, Fife KY16 9ST, UK; troy.dougherty@nuenz.com

**Keywords:** vacuum impregnation, mesoporous materials, ceria, CGO, catalysis, electron microscopy, oxidation, gas physisorption

## Abstract

Four ceria-based mesoporous oxide materials were prepared using a new vacuum impregnation (VI) templating method developed by the authors, namely, vacuum-assisted nanocasting (VAN). Two hard templates (SBA-15 and KIT-6) were employed, and products with compositions CeO_2_ and Ce_0.9_Gd_0.1_O_1.9_ (CGO) were made with each. The desired fluorite phase and composition were confirmed by powder XRD and EDS. The product structures were characterised by XRD, TEM, gas physisorption and SAXS. All products contained ordered mesoporous material in high yields. The specific surface areas (SSAs) and pore volumes of the products were determined to be high and the pore size and pore spacings related well to the templates from which the materials were synthesised. The TEM studies confirmed that the samples had a 3D pore structure, with this being the negative of the original template. The target materials were not only produced in high yields, but also displayed a porous single-crystal morphology with non-linear lattice planes. The highest SSA values and pore volumes were reported for materials impregnated using the KIT-6 template and with the CGO composition. The results suggest that VAN is an excellent and reproducible method for producing ordered mesoporous cerias and has considerable potential for wider application. All the mesoporous products showed dramatically increased reducibility in TPR experiments compared with a high-SSA nanoparticulate ceria reference. This is very promising for their potential applications in oxidation catalysts and in fuel cell components.

## 1. Introduction

Since the discovery of the MCM-41 family of mesoporous silicas by Mobil scientists in 1992 [1], there has been much interest in the synthesis of mesoporous materials. These systems can have much higher surface areas and pore volumes than disordered nanoparticles, leading to interesting physical and chemical properties. The main methods for preparing mesoporous materials are by the use of surfactants (both ionic and non-ionic) to form arrangements of micelles into which precursors can be introduced [2,3,4]; by the combustion of inorganic salts under controlled conditions [5,6,7]; and by using other mesoporous materials (usually silica or carbon) as a solid, or ‘hard’, template. The hard templating method involves the introduction of a precursor solution into the pores; thermal treatment to convert the precursor to the desired composition within the template; and finally, removal of the template by reaction, dissolution or burning to leave the mesoporous product [8,9].

Ceria has many interesting chemical properties that may be enhanced by making it mesoporous. Ceria can be readily and reversibly reduced in low partial pressures of oxygen or in chemical reactions and it has a high oxygen storage capacity, making it an ideal starting point for use in oxidation catalysts. For example, initially ceria, and now substituted cerias, have found widespread use in automotive three-way catalysts [10,11]. When ceria is partially reduced it acts as a semiconductor because of the existence of the Ce^4+^/Ce^3+^ couple [12,13]. Furthermore, if some of the Ce is aliovalently substituted by cations of suitable size and lower charge, such as Gd^3+^ or Sm^3+^, oxygen vacancies are introduced into the crystal lattice of the resulting mixed oxide, and it becomes an excellent oxygen ion conductor at high temperatures and moderate-to-high oxygen partial pressures [14,15,16,17,18]. The combination of these properties makes these materials very attractive candidates for use in intermediate temperature solid oxide fuel cells (IT-SOFCs), depending on their exact chemical composition, either in the electrolyte, where a gas-tight, pure oxygen ion conductor is required, or in the fuel anode, where the combination of oxidative catalytic activity and mixed electronic and ionic conduction in fuel atmospheres has been found to be beneficial [19,20]. Clearly, then, the preparation of mesoporous ceria and substituted cerias, in which high surface area and high porosity are added to the other properties of these materials, may be of great importance for a wide range of potential applications. It should be noted that for applications in catalysis and in fuel cell systems, structural stability at elevated temperatures would be required.

In early work, one-step methods in which a cerium salt was exposed to either an ionic [21] or a non-ionic surfactant [22] gave rise to high-surface-area mesoporous cerias. However, these were found to be structurally rather ill-defined; nanoparticulate; and therefore, thermally unstable. More ordered mesoporous structures were achieved using the hard templating, or nanocasting, method. Ordered mesoporous products of both ceria [23] and cerium gadolinium oxide (CGO, Ce_1−x_Gd_x_O_2−x/2_) [24] were prepared using silica templates and by using a solid mesoporous carbon template [25], which was itself nanocast from a mesoporous silica template. Mesoporous inorganic oxides produced from silica templates now include Cr_2_O_3_ [26,27,28,29,30,31], Co_3_O_4_ [26,29,30,31,32,33,34], In_2_O_3_ [30,31,34], MnO*_x_* [30,31,35,36,37], NiO [31,38], Fe_2_O_3_ [30,31,39], WO_3_ [40,41], ZrO_2_ [42] and CeO_2_ [23,26,30,31,40] (this method is generally restricted to materials that are not soluble in either HF or NaOH, which are the etchants typically used to remove the silica matrix, and thus, release the mesoporous product). On reviewing these oxides, it can be concluded that it is generally more difficult to obtain well-ordered mesoporous-ceria-based materials in high yield than for other oxides. One limiting factor that might be addressed is the likely difficulty with which the viscous precursor solution enters the small pores of the template. This is exemplified by SBA-15, which is a silica template with a one-dimensional arrangement (in the ***P6mm*** symmetry) of parallel cylindrical pores, which are interconnected by very fine micropores. If impregnation of these fine pores is incomplete, the final product consists of loose nanorods rather than an ordered, interconnected structure, and would therefore lack the desired structural stability [43].

In this study, we described the structural, nanoscale and redox properties of mesoporous ceria and CGO materials prepared using a new method developed by ourselves. Vacuum impregnation (VI) is used in industry, for example, for removing porosity in metallurgy [44]. In this contribution, we used VI in a vacuum-assisted nanocasting (VAN) process in order to prepare better-ordered, and therefore, more structurally stable mesoporous oxides. Using VAN reduces the reliance on the capillary action for the incorporation of the precursor solution into the mesoporous template. The method has yielded better results than the conventional incipient wetness impregnation technique (IWIT) and is quicker. Further detailed discussion and comparison of the VAN and IWIT methods are available elsewhere [45]. Mesoporous ceria and CGO were prepared from two silica templates using VAN. SBA-15 and KIT-6 were chosen as the templates because they provide two quite different pore structures to test the VAN method, are widely used and well-known structures in the field, and can be prepared reproducibly and in excellent yields. Their structures were characterised using gas physisorption, powder X-ray diffraction (XRD), small-angle X-ray scattering (SAXS) and transmission electron microscopy (TEM), and their redox properties were studied by temperature programmed reduction (TPR). TEM revealed nanobridge structures, the orientation of crystal planes over relatively large distances and curvature of the lattice planes, which may have interesting consequences in catalysis and electrocatalysis.

## 2. Materials and Methods

SBA-15 and KIT-6 were synthesised following procedures available in the literature [46,47]. In a typical synthesis of SBA-15, 2 g of the non-ionic surfactant, namely, Pluronic P123 (EO_20_PO_70_EO_20_; where EO_n_ is poly(ethylene oxide) and PO_n_ is poly(propylene oxide); Aldrich), was added to 15 cm^3^ d.i. water and 60 cm^3^ of 2 M HCl and stirred at 40 °C for 8 h. Then, 4.25 g tetraethylorthosilicate (TEOS; Fluka, London, UK, 99%) was added and stirred for 24 h at the same temperature. This mixture was hydrothermally treated at 100 °C for 24 h in a Teflon container. The resulting white solid was filtered, washed and dried. The surfactant was removed by calcining at 500 °C.

In a typical synthesis of KIT-6, 6 g of Pluronic P123 was added to 180 cm^3^ d.i. water and 50 cm^3^ of 2 M HCl and stirred at 35 °C for 6 h. Then, 6 g of n-butanol (Sigma, Gillingham, UK, 99%) was added and stirred for 1 h. A total of 12.48 g TEOS was added, and the mixture was stirred at the same temperature for 24 h, followed by a hydrothermal treatment as above. The resulting white solid was filtered, washed and dried. The surfactant was removed as above.

In a typical vacuum impregnation procedure, 0.5 g of silica was evacuated, and a precursor solution was added until the silica was fully submerged. The vacuum was released, and the precursor solution was given time to fully impregnate the template. The precursor solutions were prepared by dissolving in 0.5 cm^3^ of ethanol either 1 g of Ce(NO_3_)_3_·6H_2_O; (Acros, now Thermo-Scientific, Perth, UK, 99.5%) to prepare mesoporous CeO_2_ or 1 g of a 9:1 molar ratio of the Ce(NO_3_)_3_·6H_2_O and Gd(NO_3_)_3_·6H_2_O (Alfa Aesar, now Thermo Fisher, Heysham, UK, 99.9%) to prepare the Ce_0.9_Gd_0.1_O_1.9_ (CGO). Excess solution was decanted, and the solid material was air-dried, followed by calcination at 400 °C for 5 h and at 600 °C for 5 h (with ramp rates of 1 °C min^−1^). The silica template was removed by washing the sample three times with 2 M NaOH. Products are referred to by their composition (Ceria or CGO) and the template used to prepare them (-S for SBA-15 and -K for KIT-6).

For comparison, a nanoparticulate ceria (Ceria-X) was produced without the use of a template by calcining cerium citrate (prepared from Ce(NO_3_)_3_·6H_2_O and citric acid (Alfa Aesar, 99.5%)) as per the literature [48].

A Micrometrics ASAP 2020 instrument operating at 77 K was used to obtain Brunauer–Emmett–Teller (BET) nitrogen adsorption/desorption isotherms, specific surface areas (SSAs) and Barret–Joyner–Halenda (BJH) pore size distributions (PSDs) of all products.

XRD data were collected using a Philips PW 1710 diffractometer with Cu K*_α_* radiation (λ = 1.54 Å). Scan rates in a typical experiment were 1 °C min^−1^ over a range of 2θ = 10–80°. Peak width analysis was performed by fitting a Gaussian curve to the raw data and applying the Sherrer equation in order to obtain estimates of the average crystallite size [49].

SAXS patterns were collected using a Hecus X-ray Systems Generation 1 instrument that incorporated a modified compact. A Kratky camera with slit focussing and a PSD was used. Samples were run using a SpiCap attachment and with Cu K*_α_* radiation at 40 kV and 30 mA. Data were analysed using FindGraph 2.22 peak-fitting software.

TEM images were recorded using a JEOL JEM 2011 instrument fitted with a LaB_6_ filament and operated at 200 kV. Semi-quantitative elemental analysis by energy-dispersive X-ray spectroscopy (EDS) was performed using the Oxford Instruments X-ray analysis ISIS 300 detector mounted on the TEM instrument. The DigitalMicrograph 3.4.4 graphics suite (Gatan, Abingdon, UK) was used to analyse the TEM images and to obtain digital diffraction patterns (DDPs) from the images by a fast Fourier transform. Unless stated, TEM images were not manipulated using inverse FFT functions. Elemental analysis on bulk samples was performed by inductively coupled plasma–mass spectrometry (ICP-MS) using an Agilent (Wokingham, UK) 7500 instrument.

Temperature-programmed reduction (TPR) experiments were collected using custom-built TPR equipment coupled to a quadrupole mass spectrometer system. The sample was heated from ambient temperature to 800 °C at 5 °C min^−1^ under a flow of 5% H_2_ in Ar. Temperature-programmed desorption (TPD) experiments were performed in the same way but under a flow of pure Ar. A 50 mg sample was used in each experiment, gases were passed through water and oxygen filters prior to use, and the flow rates were 45 cm^3^ min^−1^. TPR experiments were run under identical mass spectrometer and other settings to allow for direct comparison of the spectra.

## 3. Results and Discussion

### 3.1. Powder XRD

**Figure 1:**
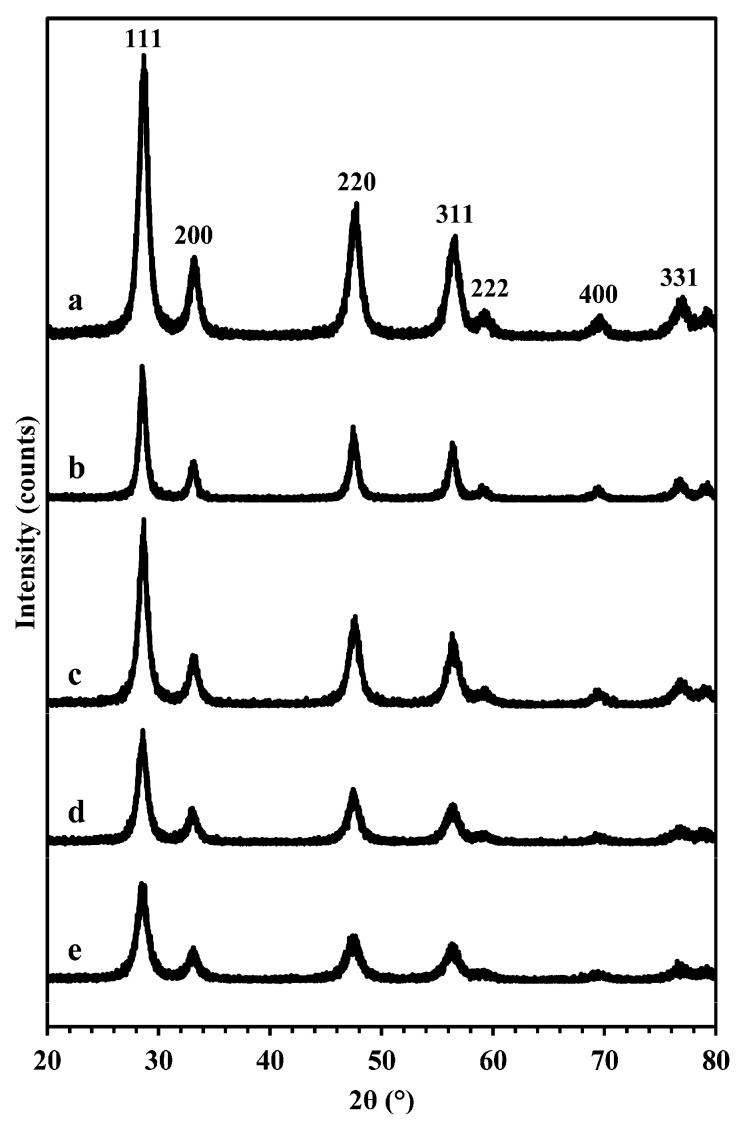
Powder XRD patterns of (a) Ceria-X reference material, (b) Ceria-S, (c) Ceria-K, (d) CGO-S and (e) CGO-K.

Powder XRD patterns for all four products—Ceria-S, Ceria-K, CGO-S and CGO-K—as well as the nanoparticulate CeO_2_, are collected in Figure 1. All patterns could be indexed to the cubic fluorite structure (Fm3m with a ~5.41 Å) expected for pure CeO_2_ and for the CGO, and there was no evidence of any impurity phases. The unit cell parameter, **a**, was 5.399 Å for the reference Ceria-X. This and the values given in Table 1 for the mesoporous products show a small general increase associated with the incorporation of the Gd into the ceria lattice, as expected from the radii of the Gd^3+^ (1.05 Å) and Ce^4+^ (0.97 Å) ions. Extensive broadening was seen in the patterns for all materials, which was indicative of small crystallites. Using the Scherrer approach, estimates of average crystallite size were found to be in the range 23.5–34.2 nm for the four products and 32.9 nm for the Ceria-X reference. The data are presented in Table 1 and are discussed below.

### 3.2. Chemical Composition

Using the EDS method in the TEM, the Ceria products were confirmed to contain Ce and O, and the CGO products contained Ce, O and Gd. The only impurity was Si, which undoubtedly remained from the templates and was detected in all samples at levels of 4.5, 6.2, 6.0 and 6.3 mol% for Ceria-S, Ceria-K, CGO-S and CGO-K, respectively. The EDS mapping showed no local concentrations of Si. Rather, it appeared to be distributed evenly throughout each sample, at least at the effective resolution of the instrument used (~5–10 nm). Molar Ce:Gd ratios were measured to be 11:1 and 10:1 for CGO-S and CGO-K, which were slightly lower than the target value of 9:1. Analysis by ICP-MS gave slightly lower values of 2 to 4 mol% Si content for these samples. The retention of some Si in products nanocast from silica-based hard templates is a known problem in the field [42]. In the current work, HF could not be used, as this would dissolve the products, and the less efficient etchant NaOH had to be employed. A detailed study of template removal would be beneficial and could cover higher concentrations of etchant, use of elevated temperatures during dissolution, further repeats of the etching process, and the use of mechanical agitation and stirring during this step.

### 3.3. Structures of SBA-15, Ceria-S and CGO-S

#### 3.3.1. Gas Physisorption

**Figure 2:**
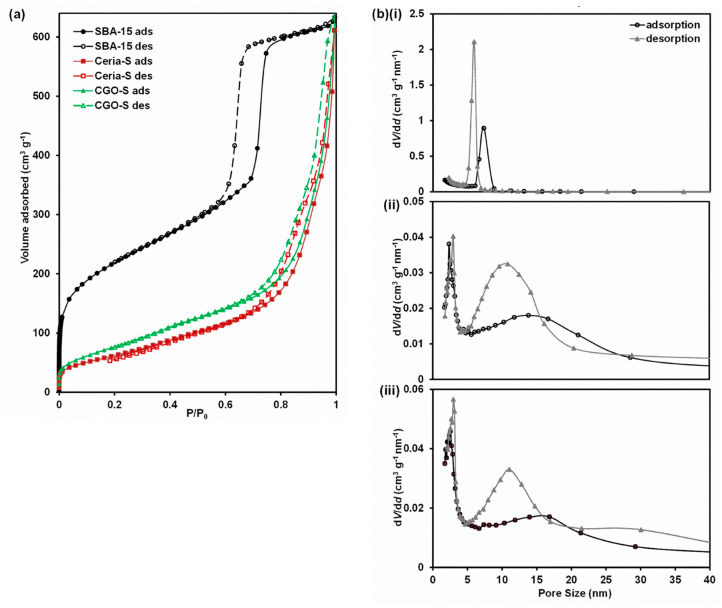
(**a**) Adsorption (ads) and desorption (des) isotherms for the SBA-15 template and the Ceria-S and CGO-S products made using it. To allow for a direct comparison, the values for the products were corrected for density (×2.72). (**b**) Pore size distributions for (**i**) the SBA-15 template and the (**ii**) Ceria-S and (**iii**) CGO-S products.

The gas adsorption–desorption isotherms and pore size distributions (PSDs) are given in Figure 2 for the SBA-15 template and the Ceria-S and CGO-S products. The isotherms for all three materials are type IV with type H3 hysteresis, which is typical of mesoporous materials where capillary condensation occurs in the mesopores. The values for SSA, specific pore volume and pore size obtained from the physisorption experiments are summarised in Table 1. For SBA-15, these relate to several batches of material and are in agreement with the literature [46,50]. Considering that the densities of ceria and CGO are around 2.72 times that of silica, the SSAs and specific pore volumes of the Ceria-S and CGO-S products indicate that very porous products were created. The SBA-15 showed narrow peaks in the PSD at 5.9–7.3 nm (determined from the adsorption and desorption branches), indicating a good-quality template.

The two products each showed a sharp peak at around 2.7 nm and a broader peak centred at around 12 nm for Ceria-S and around 14 nm for CGO-S, confirming that the products were largely mesoporous. The broad peak for both materials at around 30 nm could be assigned as interparticle porosity, and thus, was not related to the mesopore structure. The peak at around 2.7 nm could be assigned to the pores formed in the product after the removal of the walls of the template. Taking the more reliable value of interpore spacing for SBA-15 (from SAXS) of 9.3 nm and subtracting the SBA-15 pore diameter, 5.9–7.3 nm, we arrive at a value of 2.0–3.4 nm for the wall thickness in the template, which was consistent with the size of the small pores detected in the products (2.4–3.0 and 2.5–3.0 nm). This is evidence that the templating was successful and that the product had taken on the inverse (or negative) structure of the template. There are three possible explanations for the broad peaks centred on 12 and 14 nm. First, bundles of loose nanorods that existed outside the ordered mesopore structure may give rise to a broad peak at around this pore size. Second, in the mesopore product structure itself, the edges of the mesopores were accessible as long slots (in the [100] direction), unlike in SBA-15. Physisorption through these openings would be expected to give pore sizes above 2.7 nm because of their high aspect ratio. Finally, short missing sections of nanorods in the mesoporous product left voids whose diameter were the sum of two pore and one nanorod diameters, i.e., about 14–15 nm (for CGO-S, values from Table 1).

#### 3.3.2. SAXS

**Figure 3:**
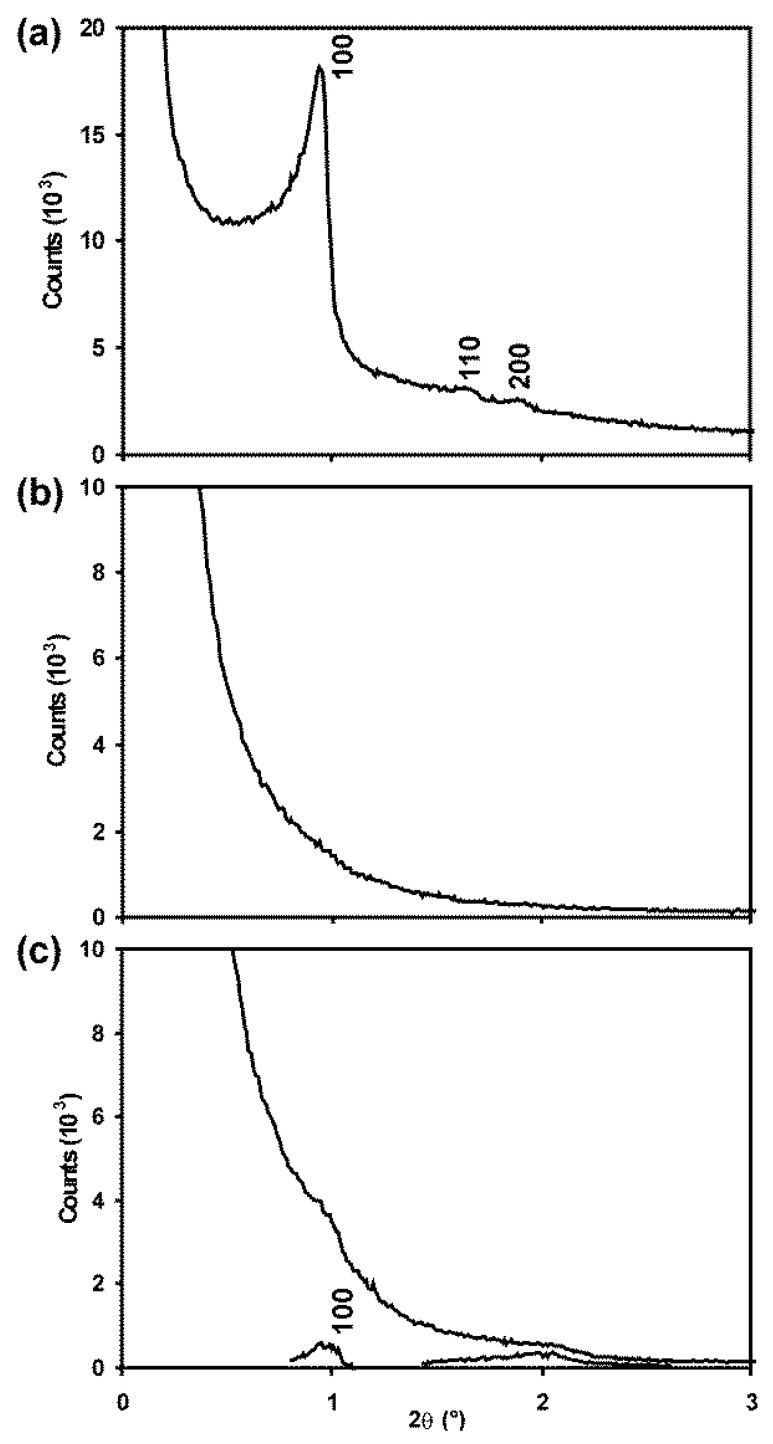
Small-angle X-ray scattering patterns for (**a**) SBA-15, (**b**) Ceria-S and (**c**) CGO-S. Miller indices related to the hexagonal pore structure are indicated.

In the SAXS patterns presented in Figure 3, diffraction peaks for SBA-15 at Miller indices of 100, 110 and 200 of the pore structure are clearly visible superimposed as shoulders on the large undiffracted instrumental peak centred at 2θ = 0°. This confirms the ordered nature of the pore structure. For Ceria-S, however, no such peaks could be identified. For CGO-S, small peaks were evident at positions that matched those of the template. These were more evident after subtracting the background (Figure 3c). The **d_100_** spacings of the pore structures are compared in Table 1 for the SBA-related materials. These would be expected to be similar, as indeed they are, if the CGO-S was successfully templated by the SBA-15. Although an inverse structure would be expected, in which voids were filled and material removed to leave pores, the size and symmetry of the repeat unit of the pore structure would be expected to remain the same in the product as it was in the template. The absence of clear SAXS peaks for Ceria-S is discussed further below.

#### 3.3.3. Electron Microscopy

**Figure 4:**
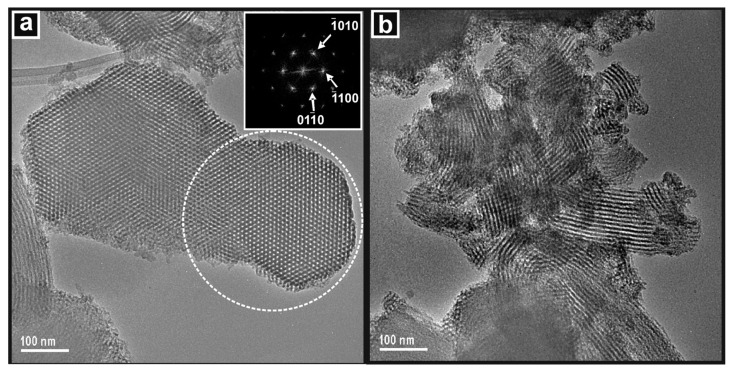
TEM images of the SBA-15 template showing (**a**) the hexagonal arrangement of cylindrical pores viewed in the [001] zone axis with DDP inset, and (**b**) the pore structure viewed in the [100] direction.

The extensive and widespread ordered pore structure of the SBA-15 template is clearly seen in the images presented in Figure 4 and by the DDP of the area indicated, which shows the hexagonal arrangement of the mesopores when viewing along the [001] zone axis. In Figure 4b, the pores are viewed along the [100] zone axis and are seen to be gently curved, which is a characteristic of SBA-15.

**Figure 5:**
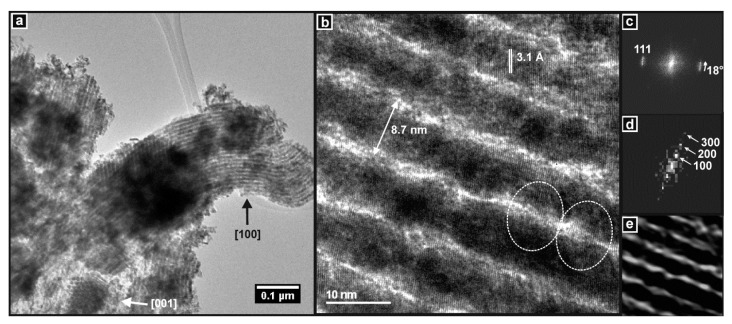
TEM images of Ceria-S. (**a**) Particles showing widespread mesoporous material, including particles viewed along the [100] and [011] zone axes of the hexagonal pore structure. (**b**) High-resolution image of mesoporous material. Bridges between the rods are circled and the interpore and interplanar distances are indicated. (**c**) DDP of (**b**) showing the 111 spot of ceria. (**d**) Enlargement of the centre of the DDP from (**c**) showing spots related to the pore structure. (**e**) Reverse Fourier transform of spots in (**d**) showing the pore structure in real space.

This curved structure is replicated in the image of the SBA-15-templated product, i.e., Ceria-S, in Figure 5a. The large agglomeration (~1 µm) in the image appears to consist largely or entirely of mesoporous particles and there are examples of both the [100] and [001] orientations (it should be kept in mind that some mesoporous particles may not appear so if they are overlapped by other particles or if their pore structure is not aligned with the electron beam of the TEM). The interpore spacing was measured directly from the images and from DDPs and gave values of 8.9–9.7 nm. This is consistent with the same dimension for the SBA-15 template obtained by the TEM and SAXS (see Table 1). The high-resolution image in Figure 5b contains several important features. First, the imaged area of the sample consists of cylindrical nanorod structures of uniform diameter separated by narrow pores. This is the inverse of the SBA-15 structure, where the pores of the template were filled with material and the walls of the template were removed to leave pores of complex shape between the nanorods. Two examples of bridges that interconnect the nanorods, and thus, hold the structure together are circled in the image. Second, the surfaces of the nanorods seemed quite rough. Both the nanorod diameter and the pore width appeared to vary slightly along the length of the nanorods. Third, the area imaged was essentially a single crystal. The crystal lattice planes were visible in the image and are seen to remain parallel across the structure and between nanorods. Therefore, the bridges between nanorods must have played an important role in achieving this long-range alignment of the lattice during crystallisation and grain growth. The DDP in Figure 5c confirmed the single-crystal nature of the imaged sample, as it contains only one pair of spots that are consistent with the 111 planes of the ceria lattice. However, and finally, these spots were, in fact, extended into short arcs—of 18° in this case—which indicates that the lattice planes gradually changed direction across the image while remaining essentially parallel. This strained crystal structure was an interesting and general feature of the mesoporous products prepared in this study.

**Figure 6:**
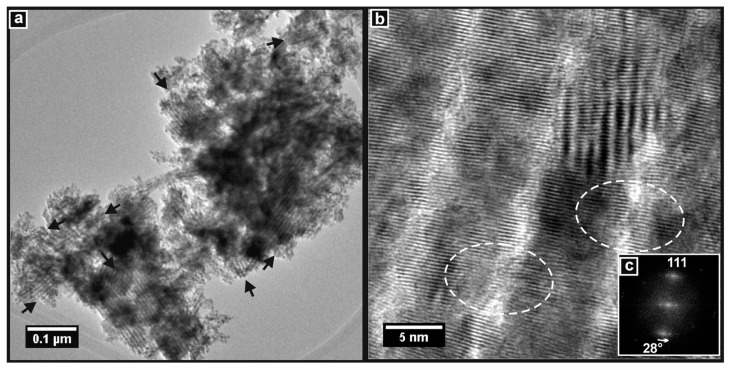
TEM images of CGO-S. (**a**) Particles showing widespread mesoporous structure (arrowed). (**b**) High-resolution image of mesoporous material. Bridges between the rods are circled. (**c**) DDP of (**b**) showing the 111 spot of CGO.

Figure 6 shows very similar features for the CGO-S material. The yield of the mesoporous material was very high and was observed throughout the sample. Some of the particles that had their pore structures aligned with the TEM beam are indicated in the image. Interpore spacings obtained from the images and DDPs agreed very well with the SAXS data for this sample and were consistent with the interpore spacings obtained for SBA-15 (Table 1). The parallel nanorods, which were separated by narrow pores and interconnected by small bridges, are seen in the high-resolution image in Figure 6b. The long-range alignment of the crystal lattice, between nanorods and across the bridges, is also evident. In the DDP of the image in Figure 6c, the spots are consistent with the 111 interplanar distance of the CGO lattice. The continuous angular variation over 28° in the position of the 111 spot indicates again, as was seen for Ceria-S, a gentle change of direction of the lattice planes across the sample. Finally, the nanorods appear to have some surface roughness, although perhaps less than for Ceria-S.

The roughness of the surface of the nanorods and the small variations in interpore distance observed for Ceria-S may have caused scattering and line broadening in the SAXS experiment, and thus, may explain the absence of peaks for Ceria-S. These structures seemed slightly smoother in CGO-S, which did give rise to peaks in the SAXS pattern. Doping ceria with Gd has been reported to aid densification during the sintering of CGO [51]. This was likely to have aided the filling of the pores of the template during crystallisation and grain growth of the CGO, resulting in the higher pore volume observed and more geometrically well-defined nanorods than in Ceria-S.

### 3.4. Structures of KIT-6, Ceria-K and CGO-K

#### 3.4.1. Gas Physisorption

**Figure 7:**
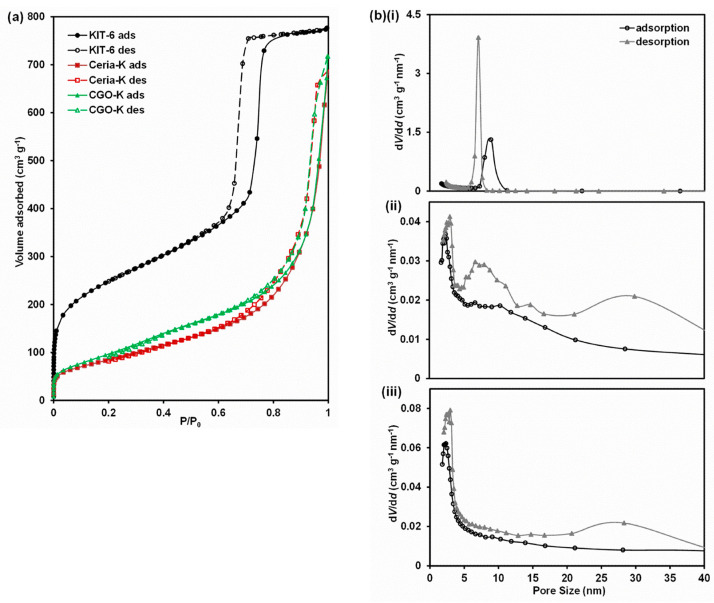
(**a**) Adsorption (ads) and desorption (des) isotherms for the KIT-6 template and the Ceria-S and CGO-S products made using it. To allow for a direct comparison, the values for the products were corrected for density (×2.72). (**b**) Pore size distributions for (**i**) the KIT-6 template and the (**ii**) Ceria-S and (**iii**) CGO-S products.

The gas adsorption–desorption isotherms and the PSD plots derived from them are presented for KIT-6, and for the Ceria-K and CGO-K made using it, in Figure 7. The isotherms for all three materials were again type IV with type H3 hysteresis, which is typical of mesoporous materials. The SSA and pore volume values were obtained from the physisorption data and are displayed in Table 1. For KIT-6, these values refer to several batches of material. As for SBA-16, the SSAs and pore volumes were very high, as expected from the literature [13,50]. For the two products, the SSAs and pore volumes were all significantly higher than for the corresponding materials prepared using the SBA-15 template. This may have at least partly been because of the SSA and pore volume being higher for KIT-6 than for SBA-15. However, it may have also been a consequence of KIT-6 having a three-dimensional, rather than a one-dimensional, pore structure like SBA-15, and thus, facilitated precursor impregnation. The PSD plots show that KIT-6 had a single narrow peak around 7 nm. Ceria-K and CGO-K showed sharp peaks around 2.5 nm; then, a poorly defined broad feature centred on about 8 nm (clearer for CGO-K); and finally, a broad peak around 25–30 nm, which could be assigned as interparticle porosity. As above, the peaks at around 2.5 nm could be attributed to the pores in the products where the template material was removed during the preparation. The poorly defined peaks around 8 nm could be explained, as above, as the interparticle porosity between disordered nanostructures external to the actual ordered mesoporous structure, as the effect of missing sections of nanorods, giving rise to relatively large voids within the mesoporous product or to adsorption through letterbox-shaped openings in the structure. It should be noted that for all products, the pores were not spherical or cylindrical, but complex shapes concentric to the nanorods and interconnected between themselves. Hence, the interpretation of the physisorption results in terms of pore shape and size was difficult.

#### 3.4.2. SAXS

**Figure 8:**
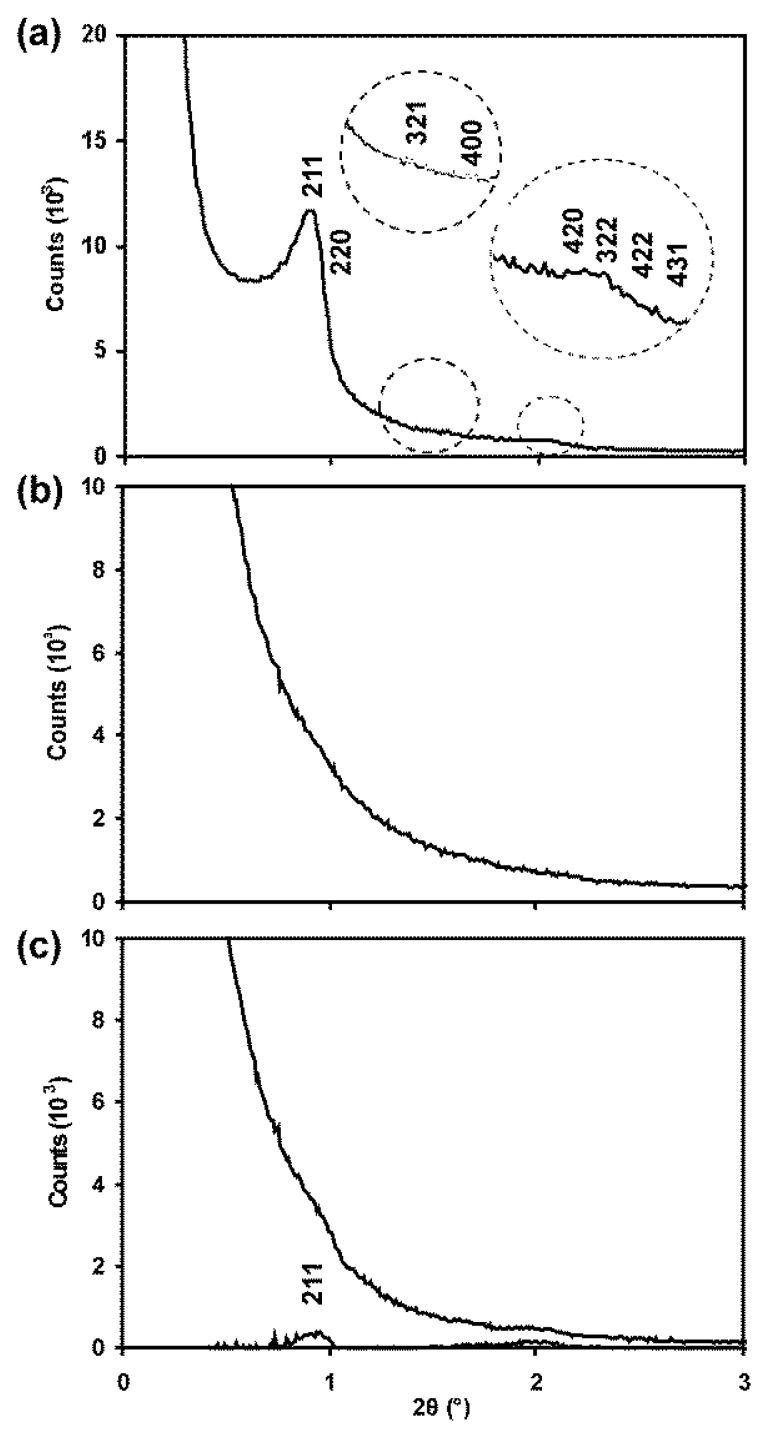
Small-angle X-ray scattering patterns for (**a**) KIT-6, (**b**) Ceria-K and (**c**) CGO-K. Miller indices related to the cubic pore structure are indicated.

In Figure 8, the SAXS pattern for KIT-6 shows one very clear peak corresponding to the 211 planes of the pore structure, a shoulder for 220 and broad features for two sets of higher-index planes. Again, the ceria product gave rise to a smooth curve with no resolvable peaks, while the CGO-K exhibited the 211 peak quite clearly, along with a broad feature around 2θ = 2°. Again, this was clearer after background subtraction. The pore spacing value, i.e., **d_220_**, for CGO-K is seen to be 10% lower than for the template, i.e., KIT-6, in Table 1.

#### 3.4.3. Electron Microscopy

**Figure 9:**
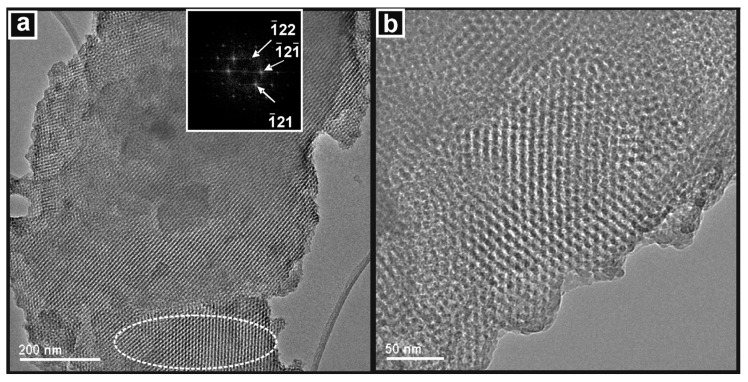
TEM images of the KIT-6 template showing (**a**) the extent of the pore structure with a DDP (inset) of a region (circled) of pores viewed in the [210] zone axis, and (**b**) a high-resolution image of the cubic pore structure.

TEM images of KIT-6 showed large particles, with some larger than 1 µm, that contained arrays of ordered mesopores across their entirety. Figure 9a shows such a particle that may be an agglomeration of several smaller ones. The pores are seen to be uniform along their length and parallel to each other. The inset DDP was taken from the circled area of the image and shows spots that can be indexed to the [211] zone axis of the cubic KIT-6 pore structure. Figure 9b is a higher-resolution image showing a region of another particle. The orientation of the pores is seen to change across the image, indicating the presence of microdomains in the pore structure.

**Figure 10:**
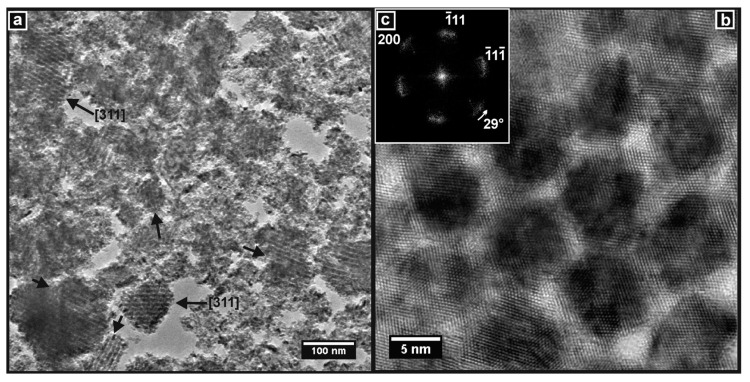
TEM images of Ceria-K. (**a**) Particles showing widespread mesoporous material, including particles viewed along the [311] zone axis of the cubic pore structure. (**b**) High-resolution image of mesoporous material viewed along the [111] zone axis of the pore structure. (**c**) DDP of (**b**) showing the full pattern of ceria viewed along the [110] crystallographic zone axis.

**Figure 11:**
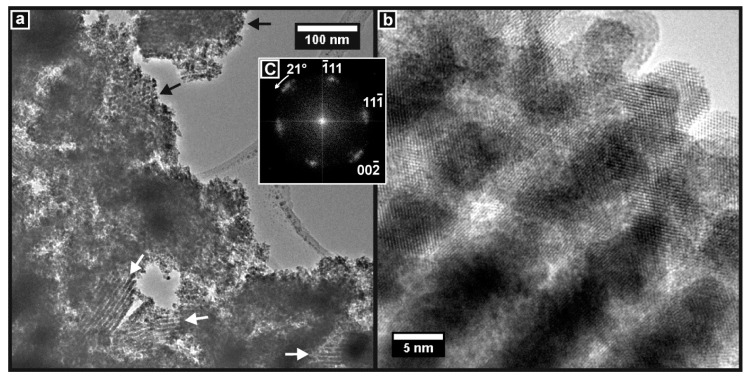
TEM images of CGO-S. (**a**) Particles showing widespread mesoporous structure (arrowed). (**b**) High-resolution image of mesoporous material showing the pore structure and crystal planes. (**c**) DDP of (**b**) showing the full pattern of CGO viewed along the [110] crystallographic zone axis.

TEM images of the Ceria-K and CGO-K products obtained using the KIT-6 template are presented in Figure 10 and Figure 11. The image in Figure 10a confirms that a very high yield of mesoporous particles was obtained, and examples are indicated in the image. Two of these had pore structures that were well-enough aligned to the TEM beam to allow them to be indexed, and they were both observed down the [311] zone axis of the (inverse) KIT-6 pore structure. Figure 10b shows a very clear high-resolution image of the mesoporous structure of Ceria-K. The concentrations of ceria material between the pores were observed as dark, roughly circular features. These were not nanorods as in SBA-15 since the direction of the wormholes in the KIT-6 template changed throughout the structure. They are better considered to be caused by the overlap—in the direction normal to the plane of the image—of nodes or junctions between (non-linear) nanorods in the inverse KIT-6 structure, which gave rise to a dark contrast in the image. These features were clearly ordered in a hexagonal arrangement with the angles between the planes measured at 60°. This indicates that the pore structure was viewed here along its [111] zone axis. In addition, the planes of the crystal lattice are clearly seen. The DDP in Figure 10c was taken from the whole image and shows a complete pattern, which could be indexed to the fluorite structure of ceria viewed along the [110] direction. Importantly, this DDP demonstrates that the crystal structure of Ceria-K was aligned across the material, with it essentially being a porous single crystal, and that the diffraction spots were, in fact, converted to short arcs by the gentle variation in the direction of the lattice planes across the sample. This same phenomenon was discussed above for Ceria-S and CGO-S.

The TEM images showed that the CGO-K material had also been successfully prepared with a widespread mesoporous structure. This is seen in Figure 11a, where the particles with aligned pore structures are identified. At high resolution, the ordered pore structure is seen to consist of essentially single-crystal CGO. Figure 11b shows such a region of the sample. The crystal lattice planes are clearly visible, and this image was used to generate the DDP in Figure 11c, which shows a complete diffraction pattern consistent with CGO viewed along the [110] zone axis. Again, the bending of the lattice planes gave rise to the arcs seen in the DDP.

### 3.5. Reduction Behaviour

**Figure 12:**
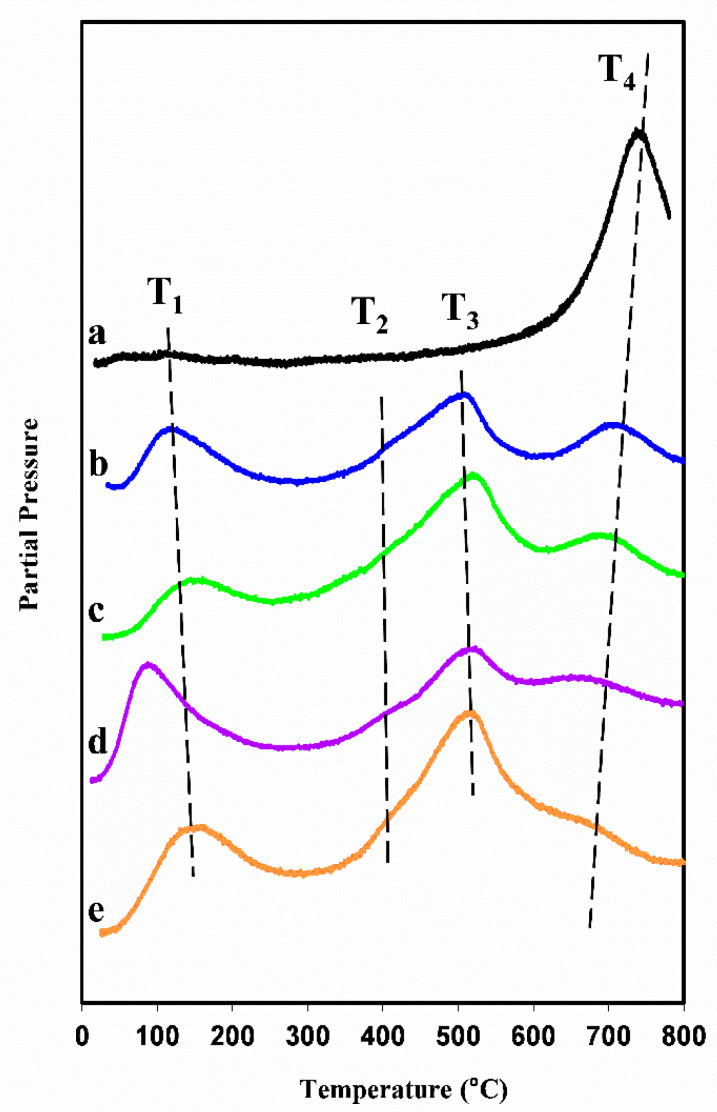
TPR spectra of (a) nanoparticulate ceria reference, (b) CGO-K, (c) Ceria-K, (d) Ceria-S and (e) CGO-S. See text for details.

The TPR spectra were obtained in flowing dilute hydrogen by recording the water signal (m/q = 18) as a function of temperature for the Ceria-X reference material and for all four products. The five spectra are presented together in Figure 12. The peaks are grouped and labelled as T_1_ to T_4_, in order of increasing temperature. The reference material exhibited a very small peak at around 100 °C (T_1_), which could be attributed to the desorption of physisorbed water from the ceria surface. The only other feature was a very large peak at 745 °C (T_4_), which was attributed to the reduction of relatively unreactive sample oxygen species by the hydrogen, which is usually assigned as bulk or lattice oxygen [52]. When comparing this spectrum with those of the four mesoporous products taken together, several important and general differences were seen.

The most important change was the occurrence of one large new peak at about 520 °C (T_3_). A second smaller new peak (T_2_) also appeared at around 430 °C as a shoulder on T_3_. Because of the size of the (T_2_ + T_3_) feature and its appearance at intermediate temperatures, it was attributed to the reduction of a large amount of reactive oxygen in the mesoporous materials. Furthermore, the fact that the T_4_ peaks were much smaller than for the reference sample indicates that the amount of relatively unreactive oxygen was much smaller. Together, these changes marked a significant shift towards active, easily available oxygen in the mesoporous samples.

The T_1_ peaks were broader and much larger than for the Ceria-X reference. These properties could be explained by the combination of up to three effects: (1) The high SSAs of the mesoporous materials allowed them to accommodate a large amount of surface water, and the mesopore network may have delayed its desorption in the transient TPR experiment to above 100 °C. (2) Ceria-based materials are known to be hygroscopic, which would further increase the amount of water on the surfaces and may delay its desorption to temperatures above 100 °C. (3) The presence of highly reactive peroxide and superoxide species on the surface of high surface area ceria has been reported [53]. The reduction of these at around 150 °C may contribute to the T_1_ peaks in the mesoporous materials. Further work is ongoing on this point.

The TPR peak positions for all samples given in Table 2 allowed us to compare the materials more closely. Peak T_1_ was discussed above and peak T_2_ is a minor shoulder whose position is hard to determine accurately. T_3_ showed little variation, which suggests that the corresponding reduction reactions were not sensitive to the composition or the mesopore structure. However, there were interesting trends for T_4_. First, T_4_ was lower for all mesoporous materials than for Ceria-X, implying that the pore structure benefitted the reduction of the bulk material. Furthermore, samples templated from KIT-6 had still higher SSAs and pore volumes than those templated from SBA-15. In addition, oxygen ion diffusion in CGO was much enhanced over that in the undoped ceria, which was likely to favour the reduction kinetics in CGO. It is interesting, then, that T_4_ was lower for the CGO samples than for the corresponding Ceria samples and lower for Ceria-K than for Ceria-S (although the same for CGO-S and CGO-K).

The marked increase in the reducibility of all four mesoporous products compared with a high-SSA ceria is of great interest in relation to their applications as oxidation catalysts. In addition, the structures with the KIT-6 morphology and CGO composition showed an additional improvement over the others, which could be related to their structural and chemical properties.

## 4. Conclusions

Four mesoporous oxide materials were prepared using a new VAN method developed by the authors. Two hard templates were employed and products with two chemical compositions were made with each. The products were characterised in detail using powder XRD, TEM, gas physisorption, SAXS and TPR studies. The results presented here suggest that the VAN method is an excellent method for producing ordered mesoporous ceria and CGO, and represents a significant improvement on established methods, such as incipient wetness impregnation, as discussed in more detail elsewhere [45]. All of the compositions and templates examined produced ordered mesoporous materials in high yields, which showed that the method was reproducible (Table 1).

The pore volumes of the products were determined to be high and the pore size and spacings related well to the templates from which the materials were synthesised. The TEM studies confirmed that the samples had a 3D structure, with this being the negative of the original template.

The materials were not only produced in high yields but also displayed a porous single-crystal morphology with non-linear lattice planes. The relatively large crystallite dimensions (22.5–34.2 nm), which were estimated from the line broadening of the XRD peaks, compared with the diameters of the individual nanostructures measured in the TEM images (~7–8 nm), support the conclusion that these materials possessed a single crystalline structure over considerable distances, both along the long axes of the nanorods and shared between neighbouring nanorods and nanostructures. This was confirmed in the diffraction patterns obtained from these TEM images. The pore walls did not exhibit large facets, as is usual for fluorite crystals. This is likely to lead to unusual, high Miller index lattice planes being exposed at the surface. The curvature of the lattice planes observed by TEM may have interesting consequences for the catalytic and electrocatalytic activity of these materials.

The highest SSA values and pore volumes were reported for materials impregnated using the KIT-6 template and using the CGO precursor solution.

The SAXS patterns showed a surprisingly low signal-to-noise ratio, which were especially poor for the Ceria materials. This may have resulted, at least in part, from unwanted scattering from the rough surfaces of the nanorods. Nevertheless, the dimensions of the pore structures were successfully obtained from the TEM images for the Ceria materials and the other products.

All of the mesoporous materials prepared using VAN showed dramatically increased reducibility in the TPR experiments compared with the high-SSA nanoparticulate ceria reference. This is very promising for their potential applications in oxidation catalysts and in SOFC components. The KIT-6 structure and CGO composition appeared to further facilitate the reduction of lattice oxygen.

The main advantage of the VAN method is that it allows for preparation in good yield and with high fidelity of products of a chosen chemical composition and nanostructure and with a very high specific surface area. High SSAs are important for active catalysts and electrocatalysts. The other unique features described here may also be significant. The curved lattice planes and the presence of potentially rare planes as facets at the surface of the nanostructures may influence the catalytic activity of the material, and the uniform size of the constituent nanostructures, and therefore, the uniform path lengths of diffusion, may be of interest for applications in catalysis, electro- and photocatalysis. We are pursuing these interesting avenues of research.

## Figures and Tables

**Table 1:** Summary of structural data for the four products: average crystallite size (**D**) from the XRD line broadening, SSA and specific pore volume (**V_p_**) from the gas sorption experiments, pore sizes (**d_p_**) from the maxima in the BJH pore size distribution plots, pore spacings from the DDPs of the TEM images (**d_TEM_**) and the SAXS patterns (**d_SAXS_**), and unit cell parameter **a** from the XRD patterns.

	D (nm)	SSA (m^2^g^−1^)	V_p_ (cm^3^g^−1^)	d_p_ (nm)	d_TEM_ (nm)	d_SAXS_ (nm)	a (Å)
SBA-15	-	800–890	1.0–1.1	5.9–7.3	7.5–8.6	9.3	-
Ceria-S	34.2	85.7	0.29	2.4–3.0, 9.6–13.8	8.9–9.7	-	5.417
CGO-S	23.5	108.6	0.32	2.5–3.0, 11–17	8.7–9.8	9.2	5.428
KIT-6	-	840–990	1.2–1.4	6.4–7.2	8.9–10.5	9.6	-
Ceria-K	24.3	114.7	0.35	2.2–3.0, ~8	8.5–9.4	-	5.412
CGO-K	22.5	137.5	0.38	2.1–2.7 ~8	9.1–9.2	8.8	5.428

**Table 2:** Positions of peaks in TPR spectra (°C).

	T_1_	T_2_	T_3_	T_4_
Ceria-X	~100	-	-	745
Ceria-S	125	-	514	712
Ceria-K	154	-	523	696
CGO-S	94	435	525	664
CGO-K	156	450	519	665

## Data Availability

Data are available on request.
